# Congratulatory Message for the New International Journal, Annals of Occupational and Environmental Medicine

**DOI:** 10.1186/2052-4374-25-3

**Published:** 2013-05-21

**Authors:** Yangho Kim

**Affiliations:** 1 President, Korean Society of Occupational and Environmental Medicine Department of Occupational and Environmental Medicine, Ulsan University Hospital, University of Ulsan College of Medicine, #290-3 Cheonha-Dong, Dong-Gu, Ulsan 682-060, Republic of Korea

## 

On behalf of all members of the Korean Society of Occupational and Environmental Medicine, I would like to extend my congratulations on the Society’s inaugural publication of a new international journal: *Annals of Occupational and Environmental Medicine*.

South Korea experienced very rapid economic development from the 1960s to 1990s, with a focus on manufacturing. The Korean Society of Occupational and Environmental Medicine was formed in 1988, when Korean people began to have serious concerns regarding workers’ health and safety. Since then, the organization has collaborated with labor and management to cope with occupational health and safety problems in Korea.

However, although Korean society has established a useful and unique occupational health and safety system that involves cooperation among labor, management, professionals, and the government, we are currently challenged by rapid changes in our industrial structure, such as increases in service industries, small businesses, and aging workers. We are also being confronted with new patterns of occupational and environmental diseases. For example, work-related musculoskeletal disorders have become prominent since the turn of the millennium, and now comprise more than half of all compensated occupational diseases. Work-related cerebrovascular and cardiovascular diseases comprising nearly a quarter of all compensated occupational diseases are important occupational health concerns, and other issues have become prominent, including mental health problems and various occupational cancers, including asbestos-related cancers. In addition, environmental health problems such as yellow dust-related health effects have become a global environmental health issue.

Thus, it is vitally important that occupational and environmental medicine researchers and professionals have an international forum through which they can share their work and experiences, especially when such information could help save or improve lives. I am sure that our new journal will significantly contribute to communicating occupational and environmental health research and practice among occupational and environmental health scientists and policy makers, allowing them to share information and learn from each other.

In this era of globalization, our new open-access online journal will strengthen the international community of occupational and environmental health.

President of the Korean Society of Occupational and Environmental Medicine.

The author, Yangho Kim, has given permission for his photo to be used as the cover page.

